# Diaqua­(1,10-phenanthrolin-2-ol)nickel(II) dinitrate

**DOI:** 10.1107/S1600536809025367

**Published:** 2009-07-08

**Authors:** Qing Yun Liu, Qi Sheng Liu, Qing Ru Zhao

**Affiliations:** a School of Chemical & Environmental Engineering, Shandong University of Science and Technology, Qingdao 266510, People’s Republic of China; b Department of Chemistry, Shandong Normal University, Jinan 250014, People’s Republic of China

## Abstract

In the mononuclear title complex, [Ni(C_12_H_8_N_2_O)_2_(H_2_O)_2_](NO_3_)_2_, the Ni^II^ ion is coordinated in a distorted octa­hedral geometry. The dihedral angle between the two mean planes defined by the phenanthroline ligands is 88.26 (6)°. Intra- and intermolecular O—H⋯O hydrogen bonds between the cation and the anions lead to the formation of a layered arrangement parallel to (010).

## Related literature

For a related crystal structure, see: Shi *et al.* (2009 ▶).
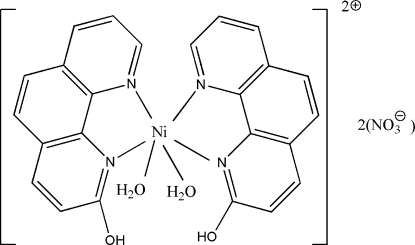


## Experimental

### 

#### Crystal data

[Ni(C_12_H_8_N_2_O)_2_(H_2_O)_2_](NO_3_)_2_
*M* *_r_* = 611.17
Monoclinic, 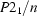

*a* = 9.6939 (16) Å
*b* = 16.386 (3) Å
*c* = 16.101 (3) Å
β = 96.126 (3)°
*V* = 2543.0 (7) Å^3^
*Z* = 4
Mo *K*α radiation
μ = 0.83 mm^−1^
*T* = 298 K
0.22 × 0.14 × 0.12 mm

#### Data collection

Bruker SMART APEX CCD diffractometer
Absorption correction: multi-scan (*SADABS*; Bruker, 1997 ▶) *T*_min_ = 0.838, *T*_max_ = 0.907
14665 measured reflections
5498 independent reflections
3422 reflections with *I* > 2σ(*I*)
*R*_int_ = 0.051

#### Refinement

*R*[*F* ^2^ > 2σ(*F* ^2^)] = 0.071
*wR*(*F* ^2^) = 0.212
*S* = 1.02
5498 reflections
373 parameters
7 restraints
H-atom parameters constrained
Δρ_max_ = 1.65 e Å^−3^
Δρ_min_ = −0.52 e Å^−3^

### 

Data collection: *SMART* (Bruker, 1997 ▶); cell refinement: *SAINT* (Bruker, 1997 ▶); data reduction: *SAINT*; program(s) used to solve structure: *SHELXTL* (Sheldrick, 2008 ▶); program(s) used to refine structure: *SHELXTL*; molecular graphics: *SHELXTL*; software used to prepare material for publication: *SHELXTL* and local programs.

## Supplementary Material

Crystal structure: contains datablocks I, global. DOI: 10.1107/S1600536809025367/bt2976sup1.cif

Structure factors: contains datablocks I. DOI: 10.1107/S1600536809025367/bt2976Isup2.hkl

Additional supplementary materials:  crystallographic information; 3D view; checkCIF report

## Figures and Tables

**Table 1:** Hydrogen-bond geometry (Å, °)

*D*—H⋯*A*	*D*—H	H⋯*A*	*D*⋯*A*	*D*—H⋯*A*
O11—H16⋯O4	0.82	1.88	2.683 (5)	166
O10—H17⋯O5	0.82	1.84	2.651 (6)	169
O5—H9⋯O8^i^	0.88	1.80	2.596 (5)	148
O5—H8⋯O1	0.89	1.88	2.757 (5)	167
O4—H5⋯O7^ii^	0.89	2.02	2.623 (5)	124
O4—H4⋯O3	0.89	1.84	2.712 (5)	165

Symmetry codes: (i) 
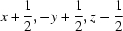
; (ii) 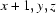.

**Fig. 1.**
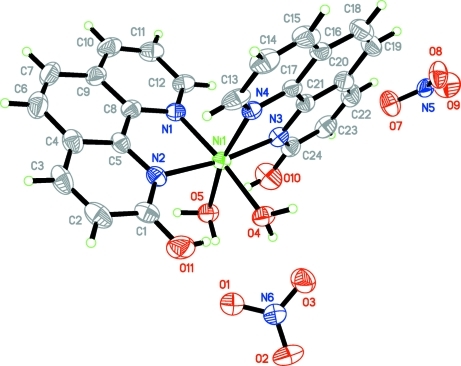
The molecular structure of the title compound showing the atom numbering scheme with thermal ellipsoids drawn at the 30% probability level.

**Fig. 2.**
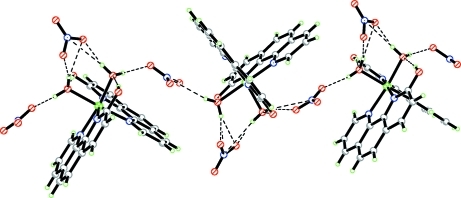
Hydrogen bonds (dashed line).

## supplementary crystallographic information

### Comment

Metal complexes containing the derivatives of 1,10-phenanthroline as ligands play a pivotal role in the area of modern coordination chemistry. A few complexes dealing with 2-hydroxyl-1,10-phenanthroline have been published (Shi *et al.*, 2009) and the interest in this area resulted in us to synthesize the title complex, and here we report its crystal structure, (I), Fig. 1.

The data of coordination bond lengths and associated angles  indicate that the Ni^II^ ion assumes a distorted octahedral geometry. All non-hydrogen atoms of the each ligand 2-hydroxyl-1,10-phenanthroline define a plane within 0.0300 Å (dealing with atom N1 plane) and 0.0200 Å (dealing with atom N3 plane) with the maximum deviation of 0.0506 (34)Å for atom N1 and of -0.0443 (33)Å for atom N4, respectively. The dihedral angle between the two planes is 88.26 (6)°, which means that two planes are almost vertical each other. In the crystal structure, there are the intramolecular O—H···O hydrogen bonds and the intermolecular O—H···O  hydrogen bonds (Table 2), and the intermolecular O—H···O hydrogen bonds that are from nitrate anion and the coordinated H_2_O lead to the formation of a one-dimensional chain as shown in Fig. 2.

### Experimental

5 ml of an aqueous solution of hydrated nickel nitrate (0.1745 g, 0.60 mmol) was added to 10 ml of a methanolic solution containing 2-hydroxyl-1,10-phenanthroline (0.1176 g, 0.60 mmol). Then NaOH (0.024 g, 0.60 mmol) was added into the mixed solution meanwhile it was stirred, and the mixture was further stirred for a few minutes. The green single crystals were obtained after the filtrate had been allowed to stand at room temperature for two weeks.

### Refinement

H atoms of water molecules were located in a difference Fourier map and refined as riding, with O—H = 0.82–0.89 Å, *U*_iso_(H) = 1.5 *U*_eq_(O). Other H atoms were placed in calculated positions, and refined as riding  with C—H = 0.93 /%A and *U*_iso_(H) = 1.2_eq_(C).

### Crystal data

**Table:**

[Ni(C_12_H_8_N_2_O)_2_(H_2_O)_2_](NO_3_)_2_	*F*(000) = 1256
*M**_r_* = 611.17	*D*_x_ = 1.596 Mg m^−^^3^
Monoclinic, *P*2_1_/*n*	Mo *K*α radiation, λ = 0.71073 Å
Hall symbol: -P 2yn	Cell parameters from 1943 reflections
*a* = 9.6939 (16) Å	θ = 2.5–21.3°
*b* = 16.386 (3) Å	µ = 0.83 mm^−^^1^
*c* = 16.101 (3) Å	*T* = 298 K
β = 96.126 (3)°	Block, green
*V* = 2543.0 (7) Å^3^	0.22 × 0.14 × 0.12 mm
*Z* = 4	

### Data collection

**Table:**

Bruker SMART APEX CCD diffractometer	5498 independent reflections
Radiation source: fine-focus sealed tube	3422 reflections with *I* > 2σ(*I*)
graphite	*R*_int_ = 0.051
φ and ω scans	θ_max_ = 27.0°, θ_min_ = 1.8°
Absorption correction: multi-scan (*SADABS*; Bruker, 1997)	*h* = −12→11
*T*_min_ = 0.838, *T*_max_ = 0.907	*k* = −20→20
14665 measured reflections	*l* = −18→20

### Refinement

**Table:**

Refinement on *F*^2^	Primary atom site location: structure-invariant direct methods
Least-squares matrix: full	Secondary atom site location: difference Fourier map
*R*[*F*^2^ > 2σ(*F*^2^)] = 0.071	Hydrogen site location: inferred from neighbouring sites
*wR*(*F*^2^) = 0.212	H-atom parameters constrained
*S* = 1.02	*w* = 1/[σ^2^(*F*_o_^2^) + (0.1199*P*)^2^] where *P* = (*F*_o_^2^ + 2*F*_c_^2^)/3
5498 reflections	(Δ/σ)_max_ = 0.008
373 parameters	Δρ_max_ = 1.65 e Å^−^^3^
7 restraints	Δρ_min_ = −0.52 e Å^−^^3^

### Special details

**Table:**

Geometry. All e.s.d.'s (except the e.s.d. in the dihedral angle between two l.s. planes) are estimated using the full covariance matrix. The cell e.s.d.'s are taken into account individually in the estimation of e.s.d.'s in distances, angles and torsion angles; correlations between e.s.d.'s in cell parameters are only used when they are defined by crystal symmetry. An approximate (isotropic) treatment of cell e.s.d.'s is used for estimating e.s.d.'s involving l.s. planes.
Refinement. Refinement of *F*^2^ against ALL reflections. The weighted *R*-factor *wR* and goodness of fit *S* are based on *F*^2^, conventional *R*-factors *R* are based on *F*, with *F* set to zero for negative *F*^2^. The threshold expression of *F*^2^ > σ(*F*^2^) is used only for calculating *R*-factors(gt) *etc*. and is not relevant to the choice of reflections for refinement. *R*-factors based on *F*^2^ are statistically about twice as large as those based on *F*, and *R*- factors based on ALL data will be even larger.

### Fractional atomic coordinates and isotropic or equivalent isotropic displacement parameters (Å^2^)

**Table:**

	*x*	*y*	*z*	*U*_iso_*/*U*_eq_	
C1	1.2334 (6)	0.2630 (3)	0.6271 (4)	0.0598 (13)	
C2	1.3351 (6)	0.2507 (4)	0.5719 (4)	0.0751 (17)	
H2	1.4202	0.2771	0.5815	0.090*	
C3	1.3101 (6)	0.2018 (4)	0.5063 (4)	0.0740 (16)	
H3	1.3787	0.1929	0.4711	0.089*	
C4	1.1785 (6)	0.1627 (3)	0.4896 (3)	0.0611 (13)	
C5	1.0844 (5)	0.1778 (3)	0.5467 (3)	0.0487 (11)	
C6	1.1434 (8)	0.1113 (4)	0.4208 (4)	0.0782 (18)	
H6	1.2091	0.0999	0.3844	0.094*	
C7	1.0151 (7)	0.0783 (3)	0.4067 (3)	0.0738 (16)	
H7	0.9938	0.0450	0.3603	0.089*	
C8	0.9476 (5)	0.1425 (3)	0.5322 (3)	0.0504 (11)	
C9	0.9129 (6)	0.0932 (3)	0.4610 (3)	0.0578 (13)	
C10	0.7784 (7)	0.0616 (3)	0.4515 (3)	0.0719 (16)	
H10	0.7510	0.0282	0.4060	0.086*	
C11	0.6874 (6)	0.0786 (3)	0.5068 (4)	0.0684 (15)	
H11	0.5973	0.0584	0.4989	0.082*	
C12	0.7310 (5)	0.1275 (3)	0.5769 (3)	0.0579 (12)	
H12	0.6682	0.1382	0.6154	0.069*	
C13	1.1424 (6)	0.0825 (3)	0.7581 (3)	0.0669 (15)	
H13	1.2077	0.1012	0.7242	0.080*	
C14	1.1690 (6)	0.0154 (3)	0.8096 (4)	0.0741 (16)	
H14	1.2541	−0.0109	0.8101	0.089*	
C15	1.0762 (7)	−0.0133 (3)	0.8591 (4)	0.0750 (17)	
H15	1.0968	−0.0596	0.8915	0.090*	
C16	0.9495 (6)	0.0264 (3)	0.8617 (3)	0.0570 (13)	
C17	0.9239 (5)	0.0947 (2)	0.8102 (3)	0.0451 (10)	
C18	0.8487 (7)	0.0017 (4)	0.9138 (3)	0.0728 (16)	
H18	0.8657	−0.0436	0.9482	0.087*	
C19	0.7298 (7)	0.0422 (4)	0.9143 (3)	0.0735 (16)	
H19	0.6651	0.0243	0.9490	0.088*	
C20	0.6984 (6)	0.1129 (3)	0.8628 (3)	0.0606 (13)	
C21	0.7968 (5)	0.1396 (3)	0.8111 (3)	0.0476 (11)	
C22	0.5746 (6)	0.1572 (4)	0.8576 (3)	0.0706 (15)	
H22	0.5048	0.1407	0.8893	0.085*	
C23	0.5542 (5)	0.2221 (3)	0.8087 (4)	0.0673 (16)	
H23	0.4717	0.2515	0.8062	0.081*	
C24	0.6594 (6)	0.2451 (3)	0.7613 (3)	0.0567 (12)	
N1	0.8571 (4)	0.1586 (2)	0.5903 (2)	0.0466 (9)	
N2	1.1087 (4)	0.2266 (2)	0.6141 (2)	0.0483 (9)	
N3	0.7805 (4)	0.2060 (2)	0.7610 (2)	0.0483 (9)	
N4	1.0152 (4)	0.1209 (2)	0.7587 (2)	0.0434 (8)	
N5	0.2324 (5)	0.1885 (3)	0.9531 (3)	0.0571 (10)	
N6	0.9923 (5)	0.4864 (3)	0.7949 (3)	0.0597 (11)	
Ni1	0.94695 (6)	0.22382 (3)	0.69161 (3)	0.0420 (2)	
O1	1.0110 (5)	0.4692 (2)	0.7221 (3)	0.0839 (12)	
O2	1.0314 (5)	0.5502 (3)	0.8263 (3)	0.0953 (14)	
O3	0.9375 (5)	0.4333 (3)	0.8351 (3)	0.0940 (14)	
O4	1.0564 (3)	0.29373 (18)	0.7868 (2)	0.0564 (9)	
H4	1.0100	0.3400	0.7938	0.085*	
H5	1.0704	0.2736	0.8387	0.085*	
O5	0.8685 (3)	0.33744 (18)	0.64926 (18)	0.0566 (8)	
H8	0.9253	0.3749	0.6742	0.085*	
H9	0.8883	0.3366	0.5970	0.085*	
O7	0.2489 (4)	0.2210 (3)	0.8873 (3)	0.0836 (13)	
O8	0.3314 (5)	0.1466 (3)	0.9880 (2)	0.0839 (12)	
O9	0.1269 (5)	0.1966 (3)	0.9843 (3)	0.1059 (16)	
O10	0.6339 (5)	0.3093 (3)	0.7152 (3)	0.1008 (14)	
H17	0.7003	0.3186	0.6891	0.151*	
O11	1.2628 (4)	0.3110 (3)	0.6896 (3)	0.0820 (11)	
H16	1.1964	0.3137	0.7171	0.123*	

### Atomic displacement parameters (Å^2^)

**Table:**

	*U*^11^	*U*^22^	*U*^33^	*U*^12^	*U*^13^	*U*^23^
C1	0.049 (3)	0.055 (3)	0.076 (4)	−0.007 (2)	0.007 (3)	0.009 (3)
C2	0.048 (3)	0.078 (4)	0.102 (5)	−0.004 (3)	0.019 (3)	0.023 (4)
C3	0.058 (4)	0.089 (4)	0.080 (4)	0.014 (3)	0.032 (3)	0.023 (3)
C4	0.066 (3)	0.061 (3)	0.057 (3)	0.011 (3)	0.012 (3)	0.016 (2)
C5	0.049 (3)	0.050 (3)	0.048 (3)	0.005 (2)	0.007 (2)	0.012 (2)
C6	0.106 (5)	0.071 (4)	0.061 (3)	0.029 (4)	0.028 (4)	0.007 (3)
C7	0.106 (5)	0.066 (3)	0.050 (3)	0.013 (3)	0.010 (3)	−0.010 (3)
C8	0.063 (3)	0.039 (2)	0.048 (2)	0.008 (2)	−0.004 (2)	0.0052 (19)
C9	0.076 (4)	0.047 (3)	0.048 (3)	0.006 (2)	−0.001 (3)	−0.001 (2)
C10	0.093 (5)	0.056 (3)	0.062 (3)	−0.003 (3)	−0.019 (3)	−0.009 (3)
C11	0.059 (3)	0.062 (3)	0.080 (4)	−0.012 (3)	−0.013 (3)	−0.004 (3)
C12	0.048 (3)	0.054 (3)	0.069 (3)	−0.009 (2)	−0.005 (2)	−0.007 (2)
C13	0.070 (4)	0.046 (3)	0.079 (3)	0.015 (2)	−0.020 (3)	−0.015 (2)
C14	0.070 (4)	0.060 (3)	0.088 (4)	0.020 (3)	−0.013 (3)	−0.010 (3)
C15	0.097 (5)	0.047 (3)	0.075 (4)	0.014 (3)	−0.019 (3)	0.010 (3)
C16	0.074 (4)	0.043 (3)	0.050 (3)	−0.011 (2)	−0.008 (2)	−0.001 (2)
C17	0.048 (3)	0.039 (2)	0.046 (2)	−0.0045 (19)	0.000 (2)	−0.0028 (19)
C18	0.080 (4)	0.067 (3)	0.068 (4)	−0.020 (3)	−0.004 (3)	0.016 (3)
C19	0.088 (5)	0.074 (4)	0.059 (3)	−0.030 (3)	0.012 (3)	0.007 (3)
C20	0.058 (3)	0.068 (3)	0.057 (3)	−0.017 (3)	0.010 (2)	−0.008 (3)
C21	0.048 (3)	0.048 (3)	0.046 (2)	−0.006 (2)	0.002 (2)	−0.006 (2)
C22	0.059 (3)	0.088 (4)	0.068 (4)	−0.014 (3)	0.024 (3)	−0.015 (3)
C23	0.048 (3)	0.078 (4)	0.077 (4)	0.009 (3)	0.009 (3)	−0.022 (3)
C24	0.052 (3)	0.059 (3)	0.059 (3)	0.002 (2)	0.009 (2)	−0.008 (2)
N1	0.042 (2)	0.045 (2)	0.052 (2)	−0.0019 (16)	0.0018 (17)	−0.0005 (17)
N2	0.040 (2)	0.053 (2)	0.053 (2)	−0.0052 (16)	0.0054 (18)	0.0069 (17)
N3	0.041 (2)	0.051 (2)	0.053 (2)	0.0049 (16)	0.0020 (18)	−0.0036 (17)
N4	0.042 (2)	0.0396 (19)	0.048 (2)	0.0030 (16)	0.0024 (17)	−0.0040 (16)
N5	0.066 (3)	0.057 (2)	0.045 (2)	0.004 (2)	−0.008 (2)	−0.0014 (19)
N6	0.054 (3)	0.055 (3)	0.069 (3)	0.006 (2)	0.002 (2)	−0.001 (2)
Ni1	0.0387 (4)	0.0426 (3)	0.0436 (3)	−0.0013 (2)	−0.0010 (2)	0.0009 (2)
O1	0.100 (3)	0.071 (3)	0.083 (3)	−0.011 (2)	0.019 (2)	−0.004 (2)
O2	0.103 (3)	0.061 (3)	0.117 (3)	−0.008 (2)	−0.010 (3)	−0.026 (2)
O3	0.101 (3)	0.082 (3)	0.108 (3)	−0.007 (3)	0.054 (3)	−0.004 (3)
O4	0.066 (2)	0.0468 (18)	0.0517 (18)	0.0024 (15)	−0.0163 (16)	0.0007 (14)
O5	0.064 (2)	0.0502 (18)	0.0517 (18)	−0.0021 (15)	−0.0100 (16)	0.0037 (14)
O7	0.067 (3)	0.110 (3)	0.072 (3)	0.006 (2)	0.001 (2)	0.024 (2)
O8	0.089 (3)	0.106 (3)	0.057 (2)	0.031 (3)	0.008 (2)	0.012 (2)
O9	0.072 (3)	0.113 (4)	0.141 (4)	0.009 (3)	0.050 (3)	−0.019 (3)
O10	0.084 (3)	0.101 (3)	0.120 (4)	0.012 (3)	0.022 (3)	0.022 (3)
O11	0.065 (3)	0.082 (3)	0.099 (3)	−0.024 (2)	0.007 (2)	−0.015 (2)

### Geometric parameters (Å, °)

**Table:**

C1—O11	1.286 (7)	C17—N4	1.347 (5)
C1—N2	1.344 (6)	C17—C21	1.437 (6)
C1—C2	1.410 (8)	C18—C19	1.332 (8)
C2—C3	1.327 (9)	C18—H18	0.9300
C2—H2	0.9300	C19—C20	1.437 (8)
C3—C4	1.427 (8)	C19—H19	0.9300
C3—H3	0.9300	C20—C22	1.397 (8)
C4—C5	1.385 (6)	C20—C21	1.402 (6)
C4—C6	1.404 (8)	C21—N3	1.354 (6)
C5—N2	1.347 (6)	C22—C23	1.327 (8)
C5—C8	1.443 (7)	C22—H22	0.9300
C6—C7	1.353 (9)	C23—C24	1.389 (7)
C6—H6	0.9300	C23—H23	0.9300
C7—C9	1.410 (7)	C24—O10	1.296 (6)
C7—H7	0.9300	C24—N3	1.338 (6)
C8—N1	1.374 (6)	N1—Ni1	2.065 (4)
C8—C9	1.413 (6)	N2—Ni1	2.106 (4)
C9—C10	1.396 (8)	N3—Ni1	2.079 (4)
C10—C11	1.348 (8)	N4—Ni1	2.072 (3)
C10—H10	0.9300	N5—O9	1.193 (6)
C11—C12	1.412 (7)	N5—O7	1.212 (5)
C11—H11	0.9300	N5—O8	1.262 (5)
C12—N1	1.320 (6)	N6—O2	1.206 (5)
C12—H12	0.9300	N6—O1	1.237 (5)
C13—N4	1.385 (6)	N6—O3	1.238 (5)
C13—C14	1.386 (7)	Ni1—O5	2.097 (3)
C13—H13	0.9300	Ni1—O4	2.107 (3)
C14—C15	1.348 (8)	O4—H4	0.8949
C14—H14	0.9300	O4—H5	0.8947
C15—C16	1.395 (8)	O5—H8	0.8914
C15—H15	0.9300	O5—H9	0.8838
C16—C17	1.399 (6)	O10—H17	0.8200
C16—C18	1.413 (7)	O11—H16	0.8200
			
O11—C1—N2	120.9 (5)	C22—C20—C21	115.9 (5)
O11—C1—C2	118.0 (5)	C22—C20—C19	125.5 (5)
N2—C1—C2	121.1 (5)	C21—C20—C19	118.6 (5)
C3—C2—C1	120.5 (6)	N3—C21—C20	124.2 (4)
C3—C2—H2	119.8	N3—C21—C17	116.8 (4)
C1—C2—H2	119.8	C20—C21—C17	119.0 (4)
C2—C3—C4	120.3 (5)	C23—C22—C20	121.7 (5)
C2—C3—H3	119.9	C23—C22—H22	119.1
C4—C3—H3	119.9	C20—C22—H22	119.1
C5—C4—C6	120.6 (5)	C22—C23—C24	118.1 (5)
C5—C4—C3	115.7 (5)	C22—C23—H23	120.9
C6—C4—C3	123.7 (6)	C24—C23—H23	120.9
N2—C5—C4	124.8 (4)	O10—C24—N3	120.0 (5)
N2—C5—C8	116.5 (4)	O10—C24—C23	115.4 (5)
C4—C5—C8	118.7 (4)	N3—C24—C23	124.6 (5)
C7—C6—C4	120.9 (6)	C12—N1—C8	117.6 (4)
C7—C6—H6	119.6	C12—N1—Ni1	129.3 (3)
C4—C6—H6	119.6	C8—N1—Ni1	113.0 (3)
C6—C7—C9	121.4 (5)	C1—N2—C5	117.7 (4)
C6—C7—H7	119.3	C1—N2—Ni1	129.1 (4)
C9—C7—H7	119.3	C5—N2—Ni1	112.8 (3)
N1—C8—C9	123.2 (5)	C24—N3—C21	115.4 (4)
N1—C8—C5	117.1 (4)	C24—N3—Ni1	131.8 (3)
C9—C8—C5	119.7 (5)	C21—N3—Ni1	112.6 (3)
C10—C9—C7	125.3 (5)	C17—N4—C13	120.2 (4)
C10—C9—C8	116.1 (5)	C17—N4—Ni1	112.7 (3)
C7—C9—C8	118.7 (5)	C13—N4—Ni1	127.0 (3)
C11—C10—C9	121.4 (5)	O9—N5—O7	121.1 (5)
C11—C10—H10	119.3	O9—N5—O8	121.3 (5)
C9—C10—H10	119.3	O7—N5—O8	117.6 (5)
C10—C11—C12	119.0 (5)	O2—N6—O1	121.5 (5)
C10—C11—H11	120.5	O2—N6—O3	121.6 (5)
C12—C11—H11	120.5	O1—N6—O3	116.8 (5)
N1—C12—C11	122.8 (5)	N1—Ni1—N4	94.29 (13)
N1—C12—H12	118.6	N1—Ni1—N3	93.77 (14)
C11—C12—H12	118.6	N4—Ni1—N3	80.16 (14)
N4—C13—C14	117.8 (6)	N1—Ni1—O5	95.45 (12)
N4—C13—H13	121.1	N4—Ni1—O5	167.48 (13)
C14—C13—H13	121.1	N3—Ni1—O5	91.36 (14)
C15—C14—C13	122.6 (5)	N1—Ni1—N2	79.68 (15)
C15—C14—H14	118.7	N4—Ni1—N2	96.29 (14)
C13—C14—H14	118.7	N3—Ni1—N2	172.35 (14)
C14—C15—C16	120.0 (5)	O5—Ni1—N2	93.17 (13)
C14—C15—H15	120.0	N1—Ni1—O4	173.62 (14)
C16—C15—H15	120.0	N4—Ni1—O4	87.43 (13)
C15—C16—C17	117.2 (5)	N3—Ni1—O4	92.58 (14)
C15—C16—C18	123.4 (5)	O5—Ni1—O4	83.74 (11)
C17—C16—C18	119.4 (5)	N2—Ni1—O4	94.04 (15)
N4—C17—C16	122.3 (4)	Ni1—O4—H4	109.3
N4—C17—C21	117.6 (4)	Ni1—O4—H5	119.5
C16—C17—C21	120.1 (4)	H4—O4—H5	103.0
C19—C18—C16	121.0 (5)	Ni1—O5—H8	106.2
C19—C18—H18	119.5	Ni1—O5—H9	100.8
C16—C18—H18	119.5	H8—O5—H9	104.7
C18—C19—C20	121.9 (5)	C24—O10—H17	109.5
C18—C19—H19	119.1	C1—O11—H16	109.5
C20—C19—H19	119.1		
			
O11—C1—C2—C3	−180.0 (6)	O11—C1—N2—Ni1	−8.2 (7)
N2—C1—C2—C3	−1.0 (8)	C2—C1—N2—Ni1	172.9 (4)
C1—C2—C3—C4	1.7 (9)	C4—C5—N2—C1	−0.3 (7)
C2—C3—C4—C5	−1.6 (8)	C8—C5—N2—C1	−177.3 (4)
C2—C3—C4—C6	178.8 (5)	C4—C5—N2—Ni1	−174.1 (3)
C6—C4—C5—N2	−179.5 (4)	C8—C5—N2—Ni1	8.9 (5)
C3—C4—C5—N2	0.9 (7)	O10—C24—N3—C21	−179.8 (5)
C6—C4—C5—C8	−2.5 (7)	C23—C24—N3—C21	0.2 (7)
C3—C4—C5—C8	177.9 (4)	O10—C24—N3—Ni1	−4.2 (7)
C5—C4—C6—C7	2.8 (8)	C23—C24—N3—Ni1	175.9 (4)
C3—C4—C6—C7	−177.7 (5)	C20—C21—N3—C24	−1.9 (6)
C4—C6—C7—C9	−0.8 (8)	C17—C21—N3—C24	178.0 (4)
N2—C5—C8—N1	−3.0 (6)	C20—C21—N3—Ni1	−178.4 (4)
C4—C5—C8—N1	179.8 (4)	C17—C21—N3—Ni1	1.5 (5)
N2—C5—C8—C9	177.6 (4)	C16—C17—N4—C13	−3.0 (6)
C4—C5—C8—C9	0.4 (6)	C21—C17—N4—C13	176.8 (4)
C6—C7—C9—C10	−179.8 (5)	C16—C17—N4—Ni1	−179.6 (3)
C6—C7—C9—C8	−1.3 (8)	C21—C17—N4—Ni1	0.2 (5)
N1—C8—C9—C10	0.8 (7)	C14—C13—N4—C17	2.2 (6)
C5—C8—C9—C10	−179.8 (4)	C14—C13—N4—Ni1	178.2 (3)
N1—C8—C9—C7	−177.8 (4)	C12—N1—Ni1—N4	87.2 (4)
C5—C8—C9—C7	1.5 (6)	C8—N1—Ni1—N4	−88.6 (3)
C7—C9—C10—C11	179.5 (5)	C12—N1—Ni1—N3	6.8 (4)
C8—C9—C10—C11	0.9 (7)	C8—N1—Ni1—N3	−169.0 (3)
C9—C10—C11—C12	−1.8 (8)	C12—N1—Ni1—O5	−85.0 (4)
C10—C11—C12—N1	1.0 (8)	C8—N1—Ni1—O5	99.3 (3)
N4—C13—C14—C15	0.3 (8)	C12—N1—Ni1—N2	−177.2 (4)
C13—C14—C15—C16	−2.1 (9)	C8—N1—Ni1—N2	7.0 (3)
C14—C15—C16—C17	1.3 (8)	C12—N1—Ni1—O4	−167.3 (10)
C14—C15—C16—C18	−177.9 (5)	C8—N1—Ni1—O4	16.9 (13)
C15—C16—C17—N4	1.3 (7)	C17—N4—Ni1—N1	−92.6 (3)
C18—C16—C17—N4	−179.5 (4)	C13—N4—Ni1—N1	91.1 (4)
C15—C16—C17—C21	−178.5 (4)	C17—N4—Ni1—N3	0.4 (3)
C18—C16—C17—C21	0.7 (6)	C13—N4—Ni1—N3	−175.8 (4)
C15—C16—C18—C19	179.4 (5)	C17—N4—Ni1—O5	48.4 (7)
C17—C16—C18—C19	0.2 (8)	C13—N4—Ni1—O5	−127.9 (6)
C16—C18—C19—C20	−0.4 (8)	C17—N4—Ni1—N2	−172.7 (3)
C18—C19—C20—C22	177.8 (5)	C13—N4—Ni1—N2	11.0 (4)
C18—C19—C20—C21	−0.3 (8)	C17—N4—Ni1—O4	93.5 (3)
C22—C20—C21—N3	2.9 (7)	C13—N4—Ni1—O4	−82.8 (4)
C19—C20—C21—N3	−178.8 (4)	C24—N3—Ni1—N1	−83.1 (4)
C22—C20—C21—C17	−177.0 (4)	C21—N3—Ni1—N1	92.7 (3)
C19—C20—C21—C17	1.2 (7)	C24—N3—Ni1—N4	−176.8 (4)
N4—C17—C21—N3	−1.2 (6)	C21—N3—Ni1—N4	−1.1 (3)
C16—C17—C21—N3	178.6 (4)	C24—N3—Ni1—O5	12.4 (4)
N4—C17—C21—C20	178.7 (4)	C21—N3—Ni1—O5	−171.8 (3)
C16—C17—C21—C20	−1.5 (6)	C24—N3—Ni1—N2	−113.9 (10)
C21—C20—C22—C23	−2.2 (8)	C21—N3—Ni1—N2	61.9 (11)
C19—C20—C22—C23	179.7 (5)	C24—N3—Ni1—O4	96.2 (4)
C20—C22—C23—C24	0.7 (8)	C21—N3—Ni1—O4	−88.0 (3)
C22—C23—C24—O10	−179.6 (5)	C1—N2—Ni1—N1	178.4 (4)
C22—C23—C24—N3	0.4 (8)	C5—N2—Ni1—N1	−8.7 (3)
C11—C12—N1—C8	0.6 (7)	C1—N2—Ni1—N4	−88.4 (4)
C11—C12—N1—Ni1	−175.0 (4)	C5—N2—Ni1—N4	84.6 (3)
C9—C8—N1—C12	−1.6 (6)	C1—N2—Ni1—N3	−150.3 (9)
C5—C8—N1—C12	179.1 (4)	C5—N2—Ni1—N3	22.6 (12)
C9—C8—N1—Ni1	174.8 (3)	C1—N2—Ni1—O5	83.4 (4)
C5—C8—N1—Ni1	−4.6 (5)	C5—N2—Ni1—O5	−103.6 (3)
O11—C1—N2—C5	179.2 (5)	C1—N2—Ni1—O4	−0.5 (4)
C2—C1—N2—C5	0.2 (7)	C5—N2—Ni1—O4	172.4 (3)

### Hydrogen-bond geometry (Å, °)

**Table:**

*D*—H···*A*	*D*—H	H···*A*	*D*···*A*	*D*—H···*A*
O11—H16···O4	0.82	1.88	2.683 (5)	166
O10—H17···O5	0.82	1.84	2.651 (6)	169
O5—H9···O8^i^	0.88	1.80	2.596 (5)	148
O5—H8···O1	0.89	1.88	2.757 (5)	167
O4—H5···O7^ii^	0.89	2.02	2.623 (5)	124
O4—H4···O3	0.89	1.84	2.712 (5)	165

Symmetry codes: (i) *x*+1/2, −*y*+1/2, *z*−1/2; (ii) *x*+1, *y*, *z*.
